# The effects of sertraline administration from adolescence to adulthood on physiological and emotional development in prenatally stressed rats of both sexes

**DOI:** 10.3389/fnbeh.2014.00260

**Published:** 2014-08-06

**Authors:** Inês Pereira-Figueiredo, Consuelo Sancho, Juan Carro, Orlando Castellano, Dolores E. López

**Affiliations:** ^1^Neuroscience Institute of Castilla y León (INCYL), University of SalamancaSalamanca, Spain; ^2^Institute of Biomedical Research of Salamanca (IBSAL), University of SalamancaSalamanca, Spain; ^3^Department of Physiology and Pharmacology, University of SalamancaSalamanca, Spain; ^4^Department of Cell Biology and Pathology, University of SalamancaSalamanca, Spain

**Keywords:** behavior, habituation, open field, restrain stress, serotonin, startle

## Abstract

Sertraline (SERT) is a clinically effective Selective Serotonin Reuptake Inhibitor (SSRI) known to increase and stabilize serotonin levels. This neurotransmitter plays an important role in adolescent brain development in both rodents and humans, and its dysregulation has been correlated with deficits in behavior and emotional regulation. Since prenatal stress may disturb serotoninergic homeostasis, the aim of this study was to examine the long-lasting effects of exposure to SERT throughout adolescence on behavioral and physiological developmental parameters in prenatally stressed Wistar rats. SERT was administered (5 mg/kg/day p.o.) from the age of 1–3 months to half of the progeny, of both sexes, of gestating dams stressed by use of a restraint (PS) or not stressed. Our data reveal that long-term SERT treatment slightly reduced weight gain in both sexes, but reversed the developmental disturbed “catch-up” growth found in PS females. Neither prenatal stress nor SERT treatment induced remarkable alterations in behavior and had no effects on mean startle reflex values. However, a sex-dependent effects of PS was found: in males the PS paradigm slightly increased anxiety-like behavior in the open field, while in females, it impaired startle habituation. In both cases, SERT treatment reversed the phenomena. Additionally, the PS animals exhibited a disturbed leukocyte profile in both sexes, which was reversed by SERT. The present findings are evidence that continuous SERT administration from adolescence through adulthood is safe in rodents and lessens the impact of prenatal stress in rats.

## Introduction

Sertraline (SERT) is a clinically effective Selective Serotonin Reuptake Inhibitor (SSRI) that increases serotonin (5-hydroxytryptamine, 5-HT) levels in the brain (Koe et al., 1983; Byerley et al., 1987; Manfridi et al., 1992) and hence plays an important role in stabilizing nervous activity.

Even though there are many children and adolescents with psychiatric disorders (Emslie and Mayes, 2001), decisions regarding the use of antidepressants in young people (such as the SSRIs) are still largely based on data from adults (Manfridi et al., 1992; de Jong et al., 2006). Efficacy measurements in humans recommend SSRIs as the initial medication of choice for young individuals in depression and for improving obsessive-compulsive disorder (OCD; Doogan and Caillard, 1988; Alderman et al., 1998; Emslie and Mayes, 2001; Moreno et al., 2006). SERT seems to be well tolerated in both children and adolescents, with adverse effects similar to those previously reported by adult patients (Alderman et al., 1998; Cook et al., 2001; Skaer et al., 2009). However, side effects with impact on later development have not yet been fully elucidated.

The serotoninergic system is highly complex, as evidenced by the great diversity of subtypes of receptors on which this neurotransmitter acts (at least 14 different subtypes) and the variety of functions regulated by each receptor subtype (O’Leary et al., 2013). 5-HT presynaptic receptors are located in the dorsal raphe nuclei (DRN) and postsynaptic 5-HT receptors (5-HTR) are largely present in the limbic system (Newport et al., 2001; Hensler, 2003; Leventopoulos et al., 2009). Thus, disturbing serotoninergic homeostasis during its development may result in a changed framework of brain connections and permanent alterations may be induced in adult behavior (Morley-Fletcher et al., 2003; Whitaker-Azmitia, 2005; Ansorge et al., 2008).

It is known that prenatal stress disturbs serotoninergic metabolism (Peters, 1986; Hayashi et al., 1998; Maccari and Morley-Fletcher, 2007) and is responsible for several psychiatric disorders and negative affective states later in life, such as anxiety and depression (Green et al., 2011). These disorders have previously been related to low 5-HT levels (Koe, 1990; Graeff, 2004; Ansorge et al., 2008), and more recently such low levels have been implicated in developmental perturbations, both in laboratory animals (Kay et al., 1998; Coe and Lubach, 2005; Götz and Stefanski, 2007) and in humans (Cottrell and Ozanne, 2008; O’Connor et al., 2013). This early type of stress can affect the loss of synapses and dendritic arborization, which normally occurs from puberty to adulthood (Barros et al., 2006; Zhang et al., 2013), and induces a decrease in the levels of 5-HT in the brains of young individuals (Hayashi et al., 1998).

It has also been suggested that anxiety and depression-like effects could be counteracted by treating prenatally stressed offspring with antidepressants that affect the serotonin system, such as SSRIs (Matar et al., 2006; Van den Hove et al., 2005). Nevertheless, few studies have addressed the impact of these treatments during the developmental stage of adolescence.

The acoustic startle reflex (ASR) and its habituation, both considered good tools for the investigation of emotional status and the brain mechanisms involved in behavioral plasticity, are often used in pharmacological animal models (Davis, 1980; Dulawa and Geyer, 2000; Quednow et al., 2004; Jensen et al., 2007). In view of the involvement of this (5-HT) transmitter system in the descending pathways modulating the startle reflex (Geyer et al., 2001; Quednow et al., 2004), it seem seems quite clear that its manipulation would alter the startle response.

Thus, to evaluate the long-lasting effects induced by both early stress and exposure to antidepressants during youth, our main goal was to determine the neurobiological changes in previously stressed rats subjected to SERT treatment. Considering during this developmental period, in humans, antidepressant treatments could last for years (O’Leary et al., 2013), SERT was given from the beginning of adolescence of the animals until the end of the experiments, when they were fully grown. Along the experiment, physiological (body weight gain, appetite, thirst, and immunological function) and behavioral measurements (anxiety-related behaviors using the open field and the ASR paradigm) were taken.

To best of our knowledge, few data on the long-term effects of pharmacological therapy with SERT during adolescence in normal (control) or previously disturbed (PS) subjects of both sexes are currently available. Thus, we hope present findings help to further our understanding of the long-term effects of antidepressants during this critical window of brain development.

## Materials and methods

### Animals

Virgin female Wistar rats CLS:WI(HAN) (*n* = 12) weighting 250 g were obtained from outbred rats from our own animal facility at the University of Salamanca. Vaginal smears were collected daily for 8 days before mating to determine the stage of the estrus cycle and the day of conception. On the day of proestrus, sexually experienced male Wistar rats were introduced for mating. The day the spermatozoa were found in the smear was designated as day 1 of pregnancy.

The animals were housed randomly and maintained under a normal 12/12 h light/dark cycle (lights on at 08:00 h) in a temperature- and humidity-controlled environment. The rats were given *ad libitum* access to food and water along the study period. The experiments were conducted in compliance with the guidelines for the use and care of laboratory animals of the European Communities Council Directive (2010/63/EU), the current Spanish legislation (RD 1201/05), and with those established by the Institutional Bioethics Committee. All efforts were made to minimize the number of animals used.

### Exposure to prenatal stress

Pregnant female rats were randomly assigned to the stress or control groups (*n* = 6 per group) and housed individually in plastic breeding cages. Stress consisted on placing the females in the third trimester of gestation (days 15–21) on transparent cylinder restrainers (7 cm diameter, 19 cm long); under a bright light directed onto the surface of the restrainer for 45 min three times a day (at 9 am, 12 pm, and 4 pm) (adapted from Lemaire et al., 2000). Control mothers were only subjected to routine changes (handling them the less as possible), as were the stressed females. All stress and control mothers delivered normally and only offspring from litters containing 9–13 pups were used in the experiments. Offspring were weighted at birth and weaned at 21 days of age, after which they were separated into group cages housing four animals of the same sex and treatment. Then, the pups were tail-marked and body weights were recorded weekly.

Thirty days after birth, pups from one of the two groups, Control vs. PS, depending on the previous treatment, were subdivided, to receive either chronic treatment with SERT (Control-SERT and PS-SERT) or not (Control, PS). This resulted in equal number of animals in each condition (*n* = 9–11 per sex and group). To avoid the effect of the dams, care was taken so that groups included no more than two pups from the same litter, in agreement with the protocols and results of previous authors (Bowman et al., 2004; Estanislau and Morato, 2006; Van den Hove et al., 2014). Additionally, a cursory analysis revealed no differences in litter sizes, the male-to-female ratio of the offspring, or pre-weaning-mortality.

### Drug administration

SERT (Besitran© Pfizer S.A. Madrid, Spain) was administered orally at a dose of 5.0 mg/kg/day in the animals’ drinking water, starting on postnatal day 30 (P30) and continuing until the end of experiments (P90). The SERT solution was prepared using filtered water as a vehicle. Liquid consumption was controlled (with calibrated bottles) and monitored every 2 days, and the dose of the drug was adjusted on the basis of the liquid consumption and animal’s weight. Freshly prepared solutions were then provided. Filtered water was given to control animals. During this period, the rats were kept in groups of four animals in polycarbonate boxes (45 cm × 30 cm × 20 cm), with unrestricted access to food. A dose of 5 mg/kg/day of SERT was chosen based on the pharmacokinetic and pharmacodynamic profiles of the drug (Byerley et al., 1987; West and Weiss, 2005; Matar et al., 2006), and to minimize the chronic side effects deriving from its administration (Greenberg et al., 2014). Its elimination half-life (approximately 26 h) makes administration once a day adequate (Doogan and Caillard, 1988; Murdoch and McTavish, 1992) and oral administration is more akin to clinical reality and provides adequate and maintained plasma levels (Murdoch and McTavish, 1992).

### Measurement of the acoustic startle response

At P30, and again at P90, all animals of both sexes were tested for the ASR. Before testing, the rats were habituated to the experimental conditions, especially regarding their introduction into the ASR apparatus. All testing was carried between 9:00 and 12:00 h, using the SR-LAB system (SDI, San Diego, CA, USA), as described by Castellano et al. (2009). The acoustic startle reflexes were measured in six identical startle-response cages (SR-LAB). Acoustic stimulus intensities and response sensitivities were calibrated (using an SR-LAB Startle Calibration System) so that they would be nearly identical in each of the six SR-LAB systems (maximum variability <1% of stimulus range and <5% of response ranges). Each testing session consisted of an acclimatization period of 5 min followed by 64 trials presented pseudo-randomly, with a mean inter-trial interval of 30 s. Sixteen of the trials involved a single-noise pulse (115 dB SPL, 20 ms of burst of white noise, used to determine the ASR), and the remaining trials consisted of 48 trials of a white noise prepulse at each of three intensity levels (65, 70, or 80 dB SPL) lasting 20 ms, followed by the startle stimulus (as above), at 50 ms inter-stimulus intervals. The session had three blocks of pulse and prepulse, with prepulse-to-pulse intervals of 50 ms. The first and last blocks were composed of pulses alone (5 in each block); the second block comprised 6 pulses alone and 9 of each of the prepulse intensities, all administered randomly. Whole body movements corresponding to startling responses were recorded and analyzed by the SR-LAB system, providing ASR latencies and amplitudes. The background noise of 65 dB SPL was generated throughout the entire session in order to avoid interference from external noise and to ensure equal experimental conditions. The percentage of habituation was calculated as the reduction in startle magnitude from block 1 to 3 of five pulses at 115 dB (%HAB = 100 × (first block – last block)/first block).

### Open field test

Spontaneous behavior was studied with the open-field (OF) test. The apparatus consisted of a round, white wooden arena (100 cm diameter, enclosed by a 50-cm-high wall), divided into an inner (7 areas subdivided into a large and a small center of 6 and 1 areas respectively) and an outer zone (12 areas adjacent to the wall). The OF apparatus was illuminated by an 80 W bulb, focused onto the field from a height of 100 cm above the floor. The behavior of each animal was studied for a period of 3 min over 3 consecutive days, and the occurrence of the following types of behavior was recorded: outer (OA) and inner (IA) exploratory activity (number of times the animal crossed into each zone and area) and rearings (Rear) (number of episodes in which the animal reared up on its back legs).

Between the introductions of each animal, the surfaces were cleaned with water and 70% ethanol. To minimize subjectivity, the behavior of the rats was recorded by two experimenters blind to the treatment conditions. All trials were performed between 11:00 and 14:00 h.

### Leukocyte counts and subpopulations

After the behavioral tests had been completed and after 60 days of SERT-treatment blood samples were taken between 09:00 and 11:00 h from a subset of animals from each group (*n* = 6 per group and sex) by cardiac puncture following intraperitoneal anesthesia with a mixture of ketamine (200 mg/kg) and xylazine (10 mg/kg). The blood was immediately transferred to EDTA (K3)-containing tubes and processed on an automatic cell counter (ADVIA 120 cytometer, Bayer, Leverkusen, Germany).

### Statistics

The variability within litters for all rats on each experimental group (given by standard deviations) was similar than the variability across litters, on all our dependent measures. Also, when data were analyzed using the effect of litter as a covariate, we found no significant effects. Thus, in the final analyses the litter as a variable was not considered and the data from each individual animal were used.

Statistical analysis were performed using the IBM^®^ SPSS^®^ software, version 20 (IBM Corp. and SPSS Inc., Chicago, IL, USA, 2011). The differences between groups were analyzed by ANOVA (one, two and three way), followed by the Fisher-PLSD-test for *post hoc* comparison if appropriate, and ANOVA mixed (or “SPLIT-PLOT”) with the Bonferroni-test. Mean differences were subjected pairwise to Student’s *t*-test, using the Levene Test for equality of variances. Pearson’s coefficient was used to determine correlations. Differences were regarded as statistically significant when *p* < 0.05.

## Results

### Effects of prenatal stress on body weight gain before adolescence

As expected, as a main effect of prenatal stress, differences were observed in the neonates’ body weights the day after delivery (P1), but only among the female pups (Table 1, *F*_1,30_ = 14.04, *p* < 0.01), since among the males the body weights of the pups were similar.

**Table 1:** **Animals’ body weight (g) before the beginning of pharmacological treatment: at birth (P1), at weaning (P21) and at 4 weeks of age (P28) (*n* = 18–20 animals per group and sex)**.

	Females		Males	
	Control	Prenatal stress	Control	Prenatal stress
**P1**	6.4 ± 1.1	5.9 ± 1.5^*aa*^	6.7 ± 1.3	6.2 ± 1.6
**P21**	40.8 ± 2.4	39.8 ± 2.1	39.5 ± 2.2	43.2 ± 2.7
**P28**	57.8 ± 2.9	64.8 ± 2.7^*a*^	68.2 ± 2.5	66.2 ± 2.8

*^a^*p* < 0.05 and ^aa^*p* < 0.01, indicate a main effect of prenatal stress (Fischer LSD test) (Mean ± standard error)*.

Body weight gain was analyzed using a three-factor ANOVA (prenatal treatment by sex by age) with repeated measures on the age factor (at this stage, three levels were used: 1, 21 and 28 days of age). Analysis of these data revealed that the prenatal treatment affected body weight gain. As the animals’ age advanced, weight by treatment (*F*_2,66_ = 3.42, *p* = 0.04) and weight by treatment by sex (*F*_2,66_ = 3.47, *p* = 0.037) interactions were found, indicating differences, in the effect of PS on growth rate, that were sex-dependent.

*Post hoc* analysis showed that even though in the males no differences were found as an effect of prenatal treatment, but in the females, these differences were present (*F*_2,30_ = 6.24, *p* = 0.005). Female pups from stressed mothers had lower birth weights (P1) than the controls, but these differences disappeared by the time of weaning (P21). However, this process changed at P28, and PS females exhibited a higher body weight than their controls (Table 1, *p* = 0.03).

At this early stage of development, sex *per se*, did not influence weight gain, and no sex differences in the neonates’ body weights were found at any point.

### Effects of prenatal stress and sertraline on body weight gain from adolescence to adulthood

**Figure 1:**
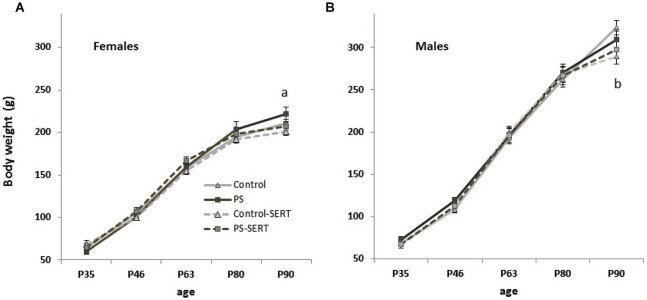
**Effects of prenatal stress and SERT treatment (5 mg/kg/day) on body weight gain (g) over time in females (A) and males (B) (*n* = 9–11 animals per group and sex)**. a, *p* < 0.05 indicates a main effect of prenatal stress; b, *p* < 0.05, indicates a main effect of SERT in Control-SERT vs. Control (Means ± standard error).

Pharmacological treatment started once adolescence had begun, and the animals’ weights were recorded weekly (for statistical analysis, five levels were used, and mean values were determined from P35 to P90). During this period, a significant overall effect of age was observed (*F*_4,73_ = 1460, *p* < 0.001), with group, *F*_16,304_ = 1.69, *p* = 0.048; sex, *F*_4,73_ = 98.8, *p* < 0.001; and group by sex, *F*_16,304_= 1.81, *p* = 0.029 interactions affecting body weight, indicating the influence of the different treatments on weight gain in each sex. As expected, there was a clear difference between the growth of males and females throughout adolescence in all experimental groups (weight by sex, *F*_4,73_ = 70.8, *p* < 0.001), with males weighting more than females (Figure 1). Accordingly, further analyses were performed separately for each sex.

At the beginning of this phase (P35) neither the prenatal manipulation nor the pharmacological treatment affected the animals’ body weights. However, at P90 (after 2 months of pharmacological treatment), differences in body weight (g) were found, both in females (*F*_3,30_ = 4.15, *p* = 0.014) and males (*F*_3,30_ = 1.8, *p* = 0.025).

Among the females, there was a main effect of group affecting weight gain with time, *F*_12,87_ = 2.12, *p* = 0.023. As depicted in Figure 1A, the animals’ growth was faster in the prenatally stressed females than in all the other experimental groups, suggesting a long-term effect of the prenatal treatment that was reversed by SERT-treatment. In fact, SERT treatment affected, at least marginally, weight gain in the females, but this was only observed at this age (P90). In males, a major effect of SERT, affecting body weight gain was observed. After 2 months of treatment it reached significance among the non-stressed males (Figure 1B).

Fluid consumption increased with age and was different between the sexes (final average: 30 ml/day for female and 35 ml/day for male rats), but not between treatments.

### ASR measures

**Figure 2:**
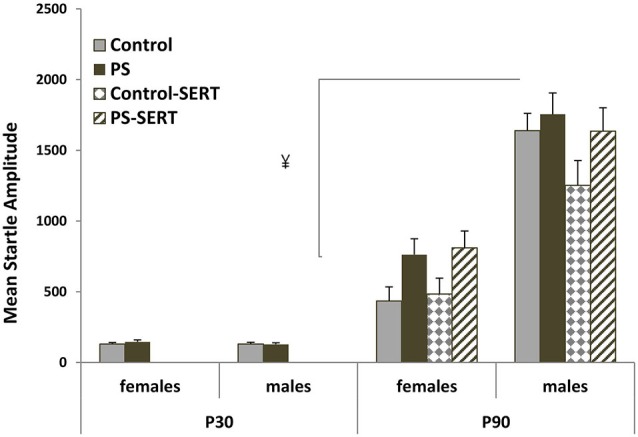
**ASR amplitude (in arbitrary units) at P30 and P90 performed in the animals of both sexes subjected or not to prenatal stress (PS and CONTROL), vs. subjected or not to prenatal stress and treated with SERT (Control-SERT and PS-SERT) (5 mg/kg/day)**. Mean values ± standard error (at P30, *n* = 18–20 animals per group and sex; at P90, *n* = 9–11 per group and sex). ¥, *p* < 0.05 indicates a main effect of sex in all experimental groups.

The startle response was first examined using a three-way mixed-design analysis of variance group by sex by age. A significant increase in startle amplitude was observed from P30 to P90 (*F*_1,77_ = 312.3, *p* < 0.001), with no group interaction, but with a strong sex interaction (*F*_1,77_ = 69.6, *p* < 0.001), marked by the differences between sexes seen at P90 (*F*_1,77_ = 58.7, *p* < 0.001), which were not seen at P30 (Figure 2).

On performing further analyses separately for each sex and age, no effects of prenatal stress at P30 were observed, whereas at P90, the greatest difference between groups were found (*F*_3,77_ = 2.67, *p* = 0.058). Although the difference did not reach statistical significance by sex, *post hoc* analysis revealed a marginal effect of prenatal stress, increasing startle amplitude in rats from both sexes regardless of SERT treatment. Additionally, SERT treatment did not induce changes in mean startle amplitude (Figure 2).

**Figure 3:**
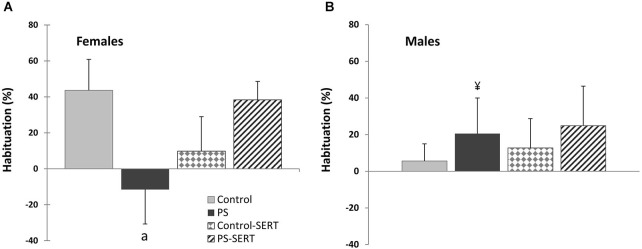
**Effects of prenatal stress and SERT treatment (5 mg/kg/day) on habituation (percentage) in females (A) and males (B) at P90**. a, *p* < 0.05, indicates a main effect of prenatal stress; ¥, *p* < 0.05, indicates a main effect of sex. Means ± standard error.

On using a block-to-block analysis to study startle amplitude, differences between the different experimental groups were observed. Startle habituation (difference between the first and the last block, expressed as percentages) was significantly different as effect of group, specifically in females (*F*_3,28_= 3.3, *p* = 0.034), probably given the deterioration of habituation found in PS females (Figure 3). Startle amplitude remained persistently high in PS females and was reversed by SERT. Given the different response to the startle test in the prenatally stressed animals (*F*_1,69_ = 3.5, *p* = 0.02), sex differences were observed specifically in PS animals (*p* = 0.03).

**Figure 4:**
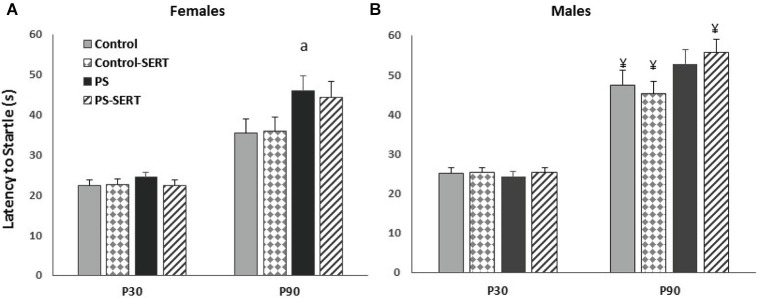
**Startle latency (stimuli 115 dB) at P30 and P90 in females (A) and males (B)**. a, *p* < 0.05 indicates a main effect of prenatal stress in females; ¥, *p* < 0.05, indicates a main effect of sex in all groups except of PS animals (Fischer LSD test). Mean values ± standard error.

Additionally, there was an overall increase in the latency to startle from P30 to P90 (*F*_1,77_ = 284.9, *p* < 0.001). Whereas at P30 no differences between groups were found; at P90, the latency to startle of PS animals was shorter than in the controls (*F*_3,77_ = 4.53, *p* = 0.006). SERT has no effect on this parameter (Figure 4), and the males showed a higher latency to startle than females (*F*_1, 77_ = 23.6, *p* < 0. 001).

Interestingly, a strong correlation was seen between ASR amplitude and its latency (*r* = 0.74, *p* < 0.001), such that the animals with higher latency responses were those with greater startle amplitudes.

### Open-field activity

Neither prenatal stress nor treatment with SERT significantly changed the activity of the animals in the OF test, although a strong influence of sex was found.

Upon analyzing the total exploratory activity (total crosses + rearings) (ANOVA with session by group by gender as within factors), no group effect was observed, but a major effect of sex was observed (*F*_1,69_ = 11.1, *p* = 0.002). Females were more active than males in all the sessions, this difference being significant in both the Control (+20.59 ± 9.4, *p* = 0.034) and PS animals (+26.1 ± 9.1, *p* < 0.01). In the case of the SERT-treated animals, the differences between sexes lost significance (Control-SERT: +5.9 ± 9.5, n.s.; PS-SERT: +14.4 ± 9.8 n.s.).

**Figure 5:**
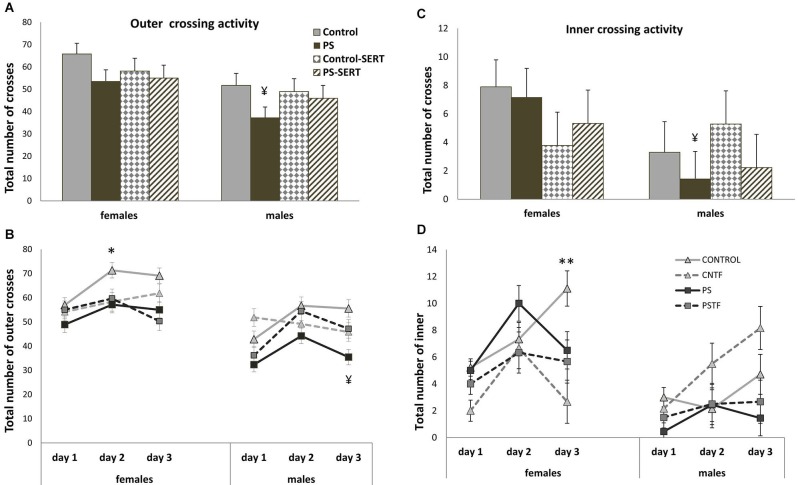
**Effects of prenatal stress and SERT treatment on the horizontal activity in the OF test (*n* = 8–9 per group and sex)**. Means ± standard error for outer crossing activity (panels **A** and **B**) and inner crossing activity (panels **C** and **D**) observed, during 3 min sessions on 3 consecutive days. No differences as effect of prenatal stress or SERT-treatment were found in outer crossing activity, *F*_3,69_ = 2.47; or inner crossing activity, *F*_3,69_ = 0.25. ¥, *p* < 0.05 indicates a main effect of sex on PS animals. *p < 0.05 and ** *p* < 0.01, indicates a significant increase on both the number of outer and inner crosses in Control females.

**Figure 6:**
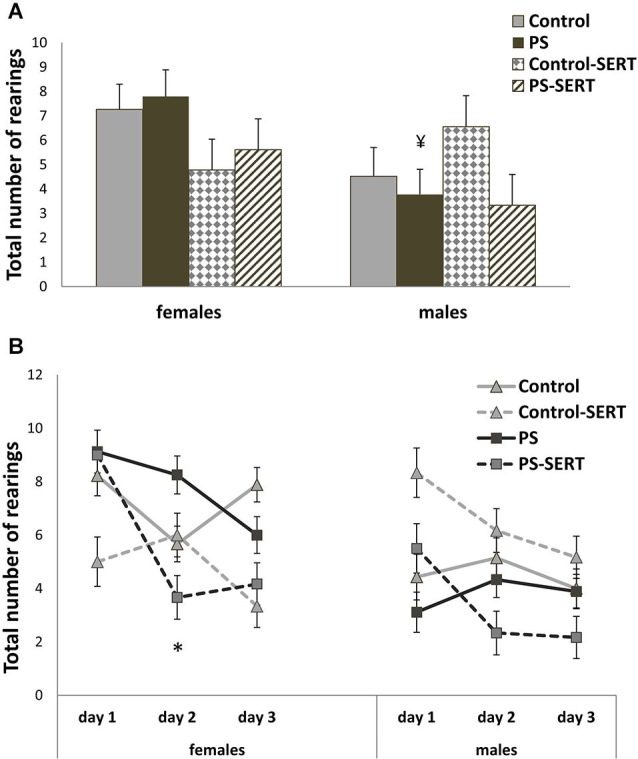
**Effects of prenatal stress and SERT treatment on the vertical activity in the OF** **test**. Number of rearings observed during 3 min sessions on 3 consecutive days (Panels **A** and **B**). Mean values ± standard error. No differences as effect of prenatal stress or SERT-treatment were found, *F*_3,69_ = 0.58, *p* > 0.05. ¥, *p* < 0.05 indicates a main effect of sex on PS animals. * *p* < 0.05, indicates a significant decrease from day 1 to day 2 of test, on the number of rearings in PS-SERT females.

A comparison of the horizontal and vertical activity performed in the sessions did not reveal differences due to stress or SERT treatment in any case (Figures 5A,C, 6A). Again, a major effect of sex (*F*_1,69_ = 10.2, *p* = 0.02; *F*_1,69_ = 3.86, *p* = 0.05; and *F*_1,69_ = 4.7, *p* = 0.035, respectively) was observed, given the overall differences between PS males and females in their willingness to engage in exploratory activity. PS males exhibited significantly less outer-field activity (Figure 5A; *F*_1,69_ = 4.23, *p* = 0.045) and inner-field activity (Figure 5C; *F*_1,69_ = 4.24, *p* = 0.038) and also fewer rearings than the females from the same group (Figure 6A; *F*_1,69_ = 7.47, *p* = 0.01).

With the exception of the Control females, in all experimental groups of both sexes the horizontal activity remained unchanged over the 3 days. In response to repeated exposure to the test, exploration in the OF increased in Control females (Figures 5B,D; both outer and inner activity). Furthermore, a different effect of the drug on inner-field activity was observed for each sex; whereas in females SERT slightly reduced inner exploration (Figures 5C,D), in males SERT treatment did not affect it, and prenatal stress seemed to be the only factor that affected such activity, and then only to a slight extent.

Moreover, the number of rearings also changed significantly over the 3 days with repeated exposure to the OF (*F*_2,68_ = 4.38, *p* = 0. 018), with no sex or group interactions (Figure 6B).

**Figure 7:**
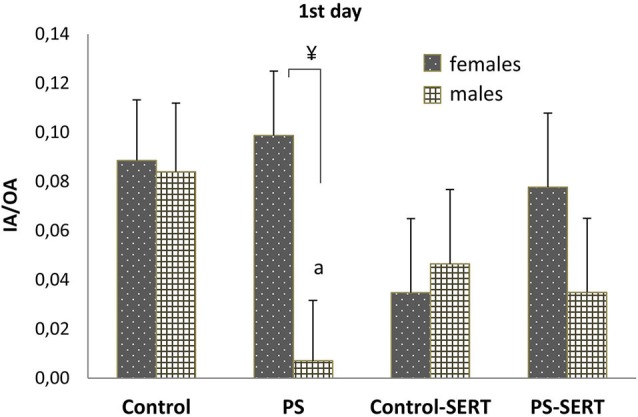
**Ratio between inner exploratory activity and outer exploratory activity (IA/OA) on the 1st day of the OF test**. a, *p* < 0.05 indicates a main effect of prenatal stress in males; ¥, *p* < 0.05 indicates a main effect of sex.

As an anxiolytic indicator, the ratio between inner-field and outer-field activity (IA/OA) was further analyzed, and no overall differences were found. However, when the analyses were split by day and sex, in males, a major effect of group was noted, specifically on the first day of the test (*F*_3,35_ = 2.98, *p* = 0.048), indicating an anxiogenic effect of prenatal stress (Figure 7, *p* = 0.043). Sex differences were also found in the PS animals (*p* = 0.014), indicating that PS increases anxiety in males, specifically, and that SERT treatment reversed it.

### Hemogram: white blood cell count

The blood leukocyte formula did not vary as a function of sex. Gestational stress compromised the immune function (Table 2). Quantitative analysis of the total of the leukocyte count revealed pronounced leukopenia in previously stressed animals and its formula was disturbed. In control animals, SERT treatment did not affect the immune response and in the stressed animals it returned pre-leukocyte failure values to normal levels. In stressed animals, differential leukocyte counts (in absolute numbers) disclosed lymphocyte levels below the normal range.

**Table 2:** **Plasma values obtained in the arterial blood in animals of both sexes (at P92)**.

Group	Leukocytes (10^3/^μl)	Neutrophils (10^3/^μl)	Lymphocytes (10^3/^μl)	Monocytes (10^3/^μl)	Eosinophils (10^3/^μl)	Basophils (10^3/^μl)
**Control**	5.45 ± 0.6	0.86 ± 0.1	4.34 ± 0.3	0.09 ± 0.01	0.12 ± 0.01	0.01 ± 0.001
**Control-SERT**	4.78 ± 0.7	1.16 ± 0.2	3.53 ± 0.6	0.07 ± 0.02	0.12 ± 0.03	0.02 ± 0.003
**PS**	1.85 ± 0.5^*aa bb*^	0.63 ± 0.1	0.93 ± 0.4^*aa b*^	0.06 ± 0.01	0.07 ± 0.02	0.003 ± 0.002^*a*^
**PS-SERT**	4.43 ± 0.6	1.00 ± 0.2	3.22 ± 0.6	0.07 ± 0.02	0.09 ± 0.02	0.01 ± 0.003
	*F* = 16.3, *p* < 0.001	*F* = 1.9, *p* = 0.14	*F* = 18.3, *p* < 0.001	*F* = 0.8, *p* = 0.49	*F* = 1.6, *p* = 0.23	*F* = 7.2, *p* < 0.01

*Means ± standard error (*n* = 6 per group). ^*a*^*p* < 0.05 and ^*aa*^*p* < 0.001 indicates a main effect of prenatal stress; ^*b*^*p* < 0.05 and ^*bb*^*p* < 0.001 indicates a main effect of SERT*.

## Discussion

The main findings of the present study highlight the importance of the exposure during development to environmental challenges affecting the serotonergic system, these effects persisting into adulthood. The sex-specific effects of prenatal stress on later physiological development and anxiety-related behaviors were remarkable. The impact of prenatal stress on immune function, later reversed by SERT administration, was observed. Importantly, it was also noted that, in rats, chronic exposure to a low dose of SERT (5 mg/kg/day) from adolescence until adulthood was safe and effective in reversing the harmful effects of prenatal stress.

### Body weight gain

Perturbations, such as maternal stress, in the uterine environment during development can permanently alter metabolism and body weight in the offspring (Pollard, 1984; Jahn et al., 1993; Lordi et al., 1997; Drago et al., 1999). The latter authors accounted for this in terms of a decrease in growth hormone (GH) and androgen production in prenatally stressed animals, which—having negative influence on growth and food intake—would lead PS animals to gain less weight.

By contrast, our results indicate that PS females increased more in weight during growth. Whereas in males no effects of early stress on weight gain were found, prenatally stressed females weighed less than their controls at birth. This difference rapidly disappeared, and was later counteracted, a trend that persisted into adulthood. Even though, this is in disagreement with earlier studies addressing prenatal stress in which PS delayed development (Pollard, 1984; Lordi et al., 1997; Berger et al., 2002), recently it has been reported in males (Chung et al., 2005; Mueller and Bale, 2006; Abe et al., 2007) and in both sexes (García-Cáceres et al., 2010). Many factors may be involved in these differences, including maternal sensitivity (Mueller and Bale, 2006), the timing of exposure to stress, or the physical properties of the stressors employed (Abe et al., 2007).

The rapid weight gain, or “catch-up” growth, reported here in PS females, that follows low birth weight has already been described in humans (Cottrell and Ozanne, 2008). According to Cottrell and Ozanne (2008) the rapid weight gain following maternal stress has important effects on later health, and so children born with abnormally low weights have been reported as being at increased risk of later obesity and related metabolic issues (Breier et al., 2001; Ozanne and Nicholas Hales, 2005). “Fetal programming” has been suggested as the origin of this (Breier et al., 2001; Tabacchi et al., 2007; Cottrell and Ozanne, 2008).

One of the biological causes of disorders involving the loss of control of the energy balance in humans is dysregulation of the serotonergic system (Kaye et al., 2005; Marston et al., 2011; Avena and Bocarsly, 2012). Brain imaging studies in patients with such disorders have uncovered alterations in 5-HT circuitry (Kaye et al., 2005; Lam et al., 2010), which have also often been described in PS animals. Accordingly, it could be speculated that such changes may at least partly affect the feeding behavior that we observed in PS females, which was reversed by SERT treatment.

SERT administration also changed weight gain and appetite. Whereas at the beginning of treatment SERT did not affect food intake, after 2 months it did. Following SERT treatment the animals exhibited a loss in body weight in comparison with their controls. This is in agreement with findings concerning food seeking behavior reported previously for adult rats, which describe the effects on weight gain of a variety of drugs able to increase the synaptic availability of 5-HT (Lucki et al., 1988; de Magalhães-Nunes et al., 2007; Mandelli et al., 2008). In adolescent male rats, the group headed by de Jong et al. (2006) also found a slight effect of another two SSRIs—fluvoxamine and paroxetine—which reduced growth. As far as we know, the present study is the first to report the long-term effects of SERT administration during development in both sexes over such a long study period (60 days).

A lack of changes in overall liquid intake was found between the groups; i.e., adding SERT at 5 mg/kg/day to the drinking water did not seem to influence the search for water. By contrast, de Magalhães-Nunes et al. (2007) reported that SERT treatment affected water and sodium intake in rats. However, those authors used a 20 mg/kg/day dose.

### Anxiety-like behavior

According to the literature, the serotonergic system modulates behavioral states (Dulawa and Geyer, 2000; Siepmann et al., 2003; Quednow et al., 2004; Iñiguez et al., 2010). However, our data show that SERT exerted no changes in mean startle reflex amplitude or in its latency in non-stressed rats.

Prenatal stress in rodents usually results in increased emotionality (Fride et al., 1986; Martí and Armario, 1998). High ASR amplitudes are found in more emotive animals (Kjær et al., 2010) and are thus considered good markers of anxiety disorders (Rasmussen et al., 2008). It is to be expected that the progeny of stressed mothers would have increased startle responses. However, the various research groups investigating the effects of PS reported no, or only marginal, differences in ASR amplitude in prenatally stressed animals (Lehmann et al., 2000; White and Birkle, 2001; Koenig et al., 2005). This is in accordance with our results, where only a slight elevation in startle amplitude was seen as an effect of prenatal stress in both sexes.

When a block-to-block analysis was performed, differences were observed. In the present study, PS males, regardless of the antidepressant treatment, exhibited a marginal elevation in the acoustically elicited startle reflex over the first few trials in comparison with the controls (data not shown). This initial increase reflects the influence of the novel, potentially aversive stimulus on the central nervous system, thus indicating PS males as being more sensitive (Meincke et al., 2004). Also, in rodents, White and Birkle (2001) reported no differences in mean startle amplitude values between PS and control progeny, except when response to novelty was tested.

By contrast, it is expected that with further stimulation, the controls would interpret the stimuli as being less relevant and the amplitude course decays in ensuing trials, revealing a process of habituation (Martí and Armario, 1998). Our data show that whereas PS males showed habituation, this was significantly impaired in PS females, SERT treatment reversing it. Acoustic startle habituation is a central point in the concept of vulnerability to stress, because it reveals the extent to which animals are able to withstand the homeostatic disturbances induced by stress over time (Koch, 1999; Meincke et al., 2004). The persistence in behavioral responses to repeated stimuli reflects difficulties in adapting to subsequent stressors and it is seen in psychotic patients (Meincke et al., 2004), meaning that SERT is an important tool for reversing stress vulnerability in PS females.

In accordance with our results, other researchers studying the physiological responses to postnatal stress in rodents found sex differences, reporting that, when stressed, males showed habituation while females showed sensitization (Chadda and Devaud, 2005; Buynitsky and Mostofsky, 2009). This sex specificity of persistently increased ASR in PS females was first reported by Hougaard et al. (2005).

Also, the latency to startle was only marginally affected by prenatal stress. PS animals from both sexes were slightly slower in becoming startled than their controls, and SERT exerted no effects. As far as we know this is the first time this effect of prenatal stress has been reported.

After the animals had been subjected to the OF test, we found that neither the prenatal stress nor SERT treatment changed locomotor activity in this test, although there was a marginal effect of early stress, anxiety increasing specifically in males. A major effect of sex was found in all parameters measured, females proving to be more active than males.

In the present work, the OF paradigm was used as a simple model to study anxiety-like behavior and locomotor activity (Durand et al., 1999; Prut and Belzung, 2003; Van den Hove et al., 2005; Rayen et al., 2011). We found sex specificity in susceptibility to early stress; the PS males exhibited increased anxiety, although this was hardly significant when compared with the PS females. PS males showed less exploratory activity and less central exploration than the females from the same group. SERT reversed it, even though it did not affect either locomotor behavior or anxiety significantly in animals from both sexes, in agreement with previous reviews about this class of antidepressants (Prut and Belzung, 2003).

The sexual dimorphism found in locomotor activity with the OF test (total crossings + rearings) has already been reported in rodents (Wakshlak and Weinstock, 1990; Pallarés et al., 2007; Duchesne et al., 2009), females being always more active than males in this kind of test. Also, vulnerability to early stress has been seen previously in the OF test (Wigger and Neumann, 1999; Nishio et al., 2001; Zueña et al., 2008). According to these authors, the effects of PS are more pronounced in males, these proving to be more emotional during the OF test. For instance, Nishio et al. (2001) reported a decrease in motor activity in neonate PS males, no effects being observed for PS females.

In the OF test, animals face contradictory motivations—the fear of an open enlightened environment, and the motivation to explore it (novelty preference) (Archer, 1973). An internal conflict underlies the motivation of the animals’ behavior (Fride et al., 1986), where high emotivity would inhibit exploration and low emotivity would facilitate it, indicating a better adaptation to the new environment (Gilad and Shiller, 1989; Durand et al., 1999). In our study, the control animals were the only ones that increased their activity with repeated exposure to the OF test.

Nonetheless, the lack of differences caused by PS observed here is in accordance with previous works (Van den Hove et al., 2005). Thus, as additional indices of anxiety-like behaviors in the OF we further determined the animals’ IA/EA activity (Archer, 1973; Durand et al., 1999; Prut and Belzung, 2003) and found that, only on the first day, prenatally stressed males exhibited a lower IA/OA ratio than controls. Again, PS males proved to be more sensitive to novelty (White and Birkle, 2001).

Additionally, although most authors defend the notion that SSRIs can normalize anxiety disorders, in rodents they behaved as anxiogenic, or anxiolytic substances, or even had no effects when the animals subjected to treatment were tested in behavioral paradigms (Durand et al., 1999; Prut and Belzung, 2003; Graeff, 2004; de Jong et al., 2006). Many variables can be invoked to account for the heterogeneity of the results, e.g., the animal model, sex, the dose or management of the SSRIs and the inclusion of a rest period. However, the timing of exposure is a determinant factor. In the present study, we tested the effects of a low dose of SERT administered during the developmental period from adolescence to adulthood, anticipating changes in serotonin-related behavior (Byerley et al., 1987; West and Weiss, 2005; Greenberg et al., 2014).

Adolescence in rodents occurs from postnatal day 28 to day 60 (Spear, 2000) and continues to be an important period of the development of the nervous system in which the serotoninergic system is still maturing (de Jong et al., 2006). Being even suggested the adolescence as a sensitive period (Eiland and Romeo, 2013; Holder and Blaustein, 2014).

Besides the changes in 5-HT transporters and their receptor activity, the release of 5-HT from the DRN in adolescent rats is increased in comparison with adult rats (de Jong et al., 2006) and the levels of 5-HT in several brain areas are also increased. As a consequence, the effects of SSRI administration during this period might be different from those elicited in adults. Actually, de Jong et al. (2006) reported that giving SSRI to adolescent rats increased anxiety, and this became apparent when they were tested as adults. In our work, we found no significant differences in anxiety-related behaviors between animals receiving SERT as compared with the controls. However, as expected, SERT played an important role in mediating the deleterious effects of prenatal stress in both sexes.

While it is known that SSRIs act mainly by binding to 5-HT transporters, then blocking 5-HT reuptake and increasing 5-HT availability, it is also known that some SSRIs have other non-specific neuropharmacological effects (Manji et al., 2001), partially acting on the inhibition of other neuroactive monoamine reuptake (Kitaichi et al., 2010) or decreasing corticotropin-releasing hormone (CRH) neuronal activity (Matar et al., 2006). More importantly, they modulate glucocorticoid receptors (GRs) activity in several brain areas (Anacker et al., 2010).

Thus, in addition to the cellular and molecular alterations induced by chronic treatment with SSRIs, reversing depressive states, other beneficial effects exist. Following the antidepressant—due increase in 5-HT concentrations in the median raphe nuclei and hippocampus, the release of neurotrophins (such as BDNF) and hippocampal neurogenesis are stimulated (Anacker et al., 2010; Willner et al., 2013). These neuroprotective and neurotrophic effects of 5-HT are known to block the damaging effects of stress on neurons (Manji et al., 2001; Morley-Fletcher et al., 2003; Hajszan et al., 2009). In fact, a role has even been suggested for antidepressants in the structural plasticity of certain cerebral areas (Pittenger and Duman, 2008). The effects of antidepressants on the hippocampus seem to be partly modulated by modifications in other brain areas that also are the sites of action of SSRIs (Castro et al., 2010). This allows at least part of the system to be restored to an almost normal state (Willner et al., 2013).

### Immunomodulatory effect

SERT has been shown to have an immunomodulatory action once it has reversed the state of leukopenia found in prenatally stressed animals. As expected, prenatal stress induced alterations in the immune function of the offsprings; this could still be seen when the immune function was determined in adulthood. A decrease was found in both total leukocyte counts in blood and in all subpopulation types, lymphocytic cells being those most affected. This has been extensively described before by authors proposing that gestational stress compromises immune function in the offspring (Kay et al., 1998; Coe and Lubach, 2005; Vanbesien-Mailliot et al., 2007; Merlot et al., 2008), with deleterious effects on leukocyte proliferation (Götz and Stefanski, 2007), and specifically on IgG levels (Sobrian et al., 1992), natural killer activity (Kay et al., 1998) and immune dysregulation by promoting pro-inflammatory and type-2 cytokine responses (Vanbesien-Mailliot et al., 2007). All these authors reported that prenatal stress altered the immune function of progeny with no, or only marginal differences, as regards sex, as was observed in the present study.

The mechanisms underlying the effects of prenatal stress on the immune system of progeny, probably result from the action of maternal stress hormones (Barbazanges et al., 1996). The increased level of glucocorticoids (GCs) reaching the developing fetus are known to affect the development of the neuroendocrine and immune systems (Kay et al., 1998; McEwen, 2008). One consequence is the HPA axis hyperactivity found in prenatally stressed rat pups that is correlated with high hormone levels, caused by an impaired feedback inhibition of GCs (glucocorticoid resistance), and these hormonal changes have been shown to regulate the magnitude and duration of the immune responses (Sobrian et al., 1992). It is known that GC binding to GRs induces their activation (transactivation). However, GRs can instead bind to transcription factors (see Anacker et al., 2010 for review), resulting in the so-called transrepression. The typical target genes of GR-mediated transrepression include inflammatory cytokines, and these latter perform the immunosuppressive action of GC hormones (Dhabhar, 2007; Anacker et al., 2010). Given such a dynamic link between both the immune and neuroendocrine systems, it could be suggested that prenatally stressed animals exhibit some impairment of the HPA axis, which would affect postnatal immune function.

Moreover, gestation is an ontogenic period during which some of the most critical events that allow normal functioning of the immune system are taking place, and, it is reasonable to assume that prenatal exposure to environmental protocols might also cause a temporary or permanent disruption in the genesis or functioning of the immune system itself (Sobrian et al., 1992). Many previous studies have shown that a critical immune organ, the thymus, is extremely sensitive to stress-responsive adrenal corticosteroids during development (Hougaard et al., 2005). Involution of the thymus, as well as lytic and apoptotic death of T cells, occurs even in adults, but is particularly pronounced in the stressed fetus (Coe and Lubach, 2005; Hougaard et al., 2005).

The long-term consequences of this early type of stress on immune function are less well known. Most of these studies reported effects in neonate or juvenile offspring. Sobrian et al. (1992) described for the first time that disruption of the immune system in PS progeny altered the postnatal response of this system to stress. Here we found that leukocyte count impairment persisted in 90-day old aged animals.

Finally, we demonstrate the central role of SERT in restoring leukocyte levels to normality. The mechanisms underlying the effects of SSRIs on the immune system under normal and pathological situations remain to be clarified (Gobin et al., 2014). Taler et al. (2008) and Gobin et al. (2014) suggested that the immunomodulatory effect of SSRIs would be related to their pro-apoptotic activity, and to their action on lymphocyte proliferation and to cytokine secretion. This may have been the case in our work as a consequence of normalizing the altered neuroendocrine function in prenatally stressed animals. Moreover, it is known that SSRIs improve 5-HT levels and can act directly on hippocampal cells, changing GR binding and regulating GRmRNA expression in neuronal cells (Smythe et al., 1994; Anacker et al., 2010).

As far as we know this is the first work to study the impact of SERT in reversing the adverse effects of prenatal stress on immune competence. The low doses used here may have been crucial, since Sacre et al. (2010), for instance, reported that a low dose of another SSRI (fluoxetine) was needed to produce significant changes in autoimmune disease and cancer.

In conclusion, our results contribute to the general knowledge about the beneficial effects of SERT, a drug known to exert anti-depressive effects, reversing early stress-associated impairments. Such beneficial effects are therefore of considerable interest in clinical practice. Also, the central role of sex susceptibility to maternal restriction stress is highlighted. The sex-dependent effects reported here could be due to the sex-specific timing of developmental processes during gestation (Roussel et al., 2005). This should be addressed in future studies.

## Conflict of interest statement

The authors declare that the research was conducted in the absence of any commercial or financial relationships that could be construed as a potential conflict of interest.
